# Root architecture plasticity in response to endoparasitic cyst nematodes is mediated by damage signaling

**DOI:** 10.1111/nph.18570

**Published:** 2022-12-01

**Authors:** Nina Guarneri, Jaap‐Jan Willig, Mark G. Sterken, Wenkun Zhou, M. Shamim Hasan, Letia Sharon, Florian M. W. Grundler, Viola Willemsen, Aska Goverse, Geert Smant, Jose L. Lozano‐Torres

**Affiliations:** ^1^ Laboratory of Nematology Wageningen University & Research 6708 PB Wageningen the Netherlands; ^2^ Laboratory of Molecular Biology, Cluster of Plant Developmental Biology Wageningen University & Research 6708 PB Wageningen the Netherlands; ^3^ State Key Laboratory of Plant Physiology and Biochemistry College of Biological Sciences, China Agricultural University Beijing 100193 China; ^4^ Institute of Crop Science and Resource Conservation (INRES), Molecular Phytomedicine University of Bonn 53115 Bonn Germany

**Keywords:** *Arabidopsis thaliana*, auxin, damage, ERF109, *Heterodera schachtii*, jasmonates, root architecture, root plasticity

## Abstract

- Plant root architecture plasticity in response to biotic stresses has not been thoroughly investigated. Infection by endoparasitic cyst nematodes induces root architectural changes that involve the formation of secondary roots at infection sites. However, the molecular mechanisms regulating secondary root formation in response to cyst nematode infection remain largely unknown.
- We first assessed whether secondary roots form in a nematode density‐dependent manner by challenging wild‐type Arabidopsis plants with increasing numbers of cyst nematodes (*Heterodera schachtii*). Next, using jasmonate‐related reporter lines and knockout mutants, we tested whether tissue damage by nematodes triggers jasmonate‐dependent secondary root formation. Finally, we verified whether damage‐induced secondary root formation depends on local auxin biosynthesis at nematode infection sites.
- Intracellular host invasion by *H. schachtii* triggers a transient local increase in jasmonates, which activates the expression of ERF109 in a COI1‐dependent manner. Knockout mutations in COI1 and ERF109 disrupt the nematode density‐dependent increase in secondary roots observed in wild‐type plants. Furthermore, ERF109 regulates secondary root formation upon *H. schachtii* infection via local auxin biosynthesis.
- Host invasion by *H. schachtii* triggers secondary root formation via the damage‐induced jasmonate‐dependent ERF109 pathway. This points at a novel mechanism underlying plant root plasticity in response to biotic stress.

## Introduction

Plants utilize root plasticity as a key strategy to survive in a changing soil environment. Remodeling of root systems allows plants to cope with nutrient deficiencies, drought, salinity, and other abiotic stresses (Koevoets *et al*., 2016). However, little is known about root architecture plasticity in response to soil‐borne biotic stresses. Infections by cyst nematodes are known to induce elaborate root architectural changes in host plants. Secondary roots form locally at cyst nematode infection sites (Grymaszewska & Golinowski, 1991; Goverse *et al*., 2000; Lee *et al*., 2011). Furthermore, the ability to form secondary roots in response to nematode infection can result in better maintenance of shoot growth in some potato and soybean cultivars (Trudgill & Cotes, 1983; Miltner *et al*., 1991). Nevertheless, the molecular mechanisms regulating secondary root formation in response to belowground herbivory are not well‐understood.

Cyst nematodes are microscopic root endoparasites that cause large agricultural losses world‐wide. These nematodes can persist in the soil in a dormant state for many years (Jones *et al*., 2013). Exudates from host roots trigger hatching of dormant second‐stage juveniles (J2s) and guide their migration to the root surface. Here, the J2s penetrate the root epidermis of the differentiation or mature root zone by piercing plant cell walls with their needle‐like oral stylet and by secreting plant cell wall degrading enzymes (Bohlmann & Sobczak, 2014). Subsequently, juveniles migrate intracellularly within the cortex, leaving behind a trail of destruction (Wyss & Zunke, 1986; Grundler *et al*., 1994). Plant cell wall fragments released during nematode migration can act as damage‐associated molecular patterns triggering defense signaling in the host (Shah *et al*., 2017). Nematode migration also activates biosynthesis and signaling of the defense hormone jasmonate (JA) (Kammerhofer *et al*., 2015). Upon successful arrival at the vascular cylinder, cyst nematodes utilize stylet‐secreted effectors to manipulate plant developmental pathways to transform host cells into permanent feeding sites (Gheysen & Mitchum, 2011). Together with permanent feeding site development, multiple *de novo* formed secondary roots emerge in clusters at nematode infection sites (Grymaszewska & Golinowski, 1991; Goverse *et al*., 2000; Lee *et al*., 2011).

Nematode feeding sites are characterized by the local accumulation of the plant hormone auxin (Karczmarek *et al*., 2004; Grunewald *et al*., 2009). Auxin transport and auxin‐insensitive Arabidopsis mutants infected by cyst nematodes show smaller females and smaller feeding sites, respectively (Goverse *et al*., 2000; Grunewald *et al*., 2009). Additionally, auxin is an important regulator of secondary root formation. Oscillations of auxin maxima at the root tip determine the formation of lateral roots in a regularly spaced pattern along the primary root (Fukaki & Tasaka, 2009). However, these oscillations are not required for the *de novo* formation of secondary roots. Ectopic induction of local auxin biosynthesis in pericycle cells via an inducible promoter is sufficient to trigger *de novo* secondary root formation (Dubrovsky *et al*., 2008). Auxin accumulation in multiple neighboring pericycle cells can lead to the formation of secondary root clusters (Dubrovsky *et al*., 2008). The spatial co‐occurrence of nematode feeding sites and secondary root clusters often corresponds to overlapping regions of auxin accumulation (Karczmarek *et al*., 2004; Absmanner *et al*., 2013). This suggests that secondary roots could be induced as the sole consequence of the auxin that accumulates during nematode feeding site development (Goverse *et al*., 2000). Alternatively, damage caused by nematode infection might also lead to local auxin accumulation and secondary root formation.

Tissue damage triggers auxin accumulation and *de novo* root formation via the JA‐dependent ERF109 transcription factor in leaf explants (Liu *et al*., 2014; Chen *et al*., 2016; Hu & Xu, 2016; Zhang *et al*., 2019). Herein, JA accumulates at the site of wounding within a few hours of leaf detachment and triggers expression of the transcription factor ERF109 via the JA receptor COI1. ERF109 binds to the promoter of the auxin biosynthesis gene ASA1, which induces root formation in a process referred to as *de novo* root organogenesis. Direct interaction of JAZ proteins inhibits ERF109 expression in a negative feedback loop to avoid wound hypersensitivity (Zhang *et al*., 2019). Sterile mechanical injury in primary roots of Arabidopsis can trigger auxin accumulation at the wounding site and subsequent secondary root formation (Sheng *et al*., 2017). However, whether this occurs via the same damage signaling pathway as *de novo* root organogenesis from leaf explants is unknown. Furthermore, mechanical injury is an artificial condition, and therefore it remains unclear whether the JA‐dependent ERF109 pathway is involved in the regulation of secondary root formation also upon naturally occurring damage by herbivory or pathogen penetration.

Previously, we showed that components of the JA‐dependent ERF109 pathway are induced by root‐knot nematode (*Meloidogyne* spp.) infection (Zhou *et al*., 2019). Differently from cyst nematodes, root‐knot nematodes penetrate roots at the elongation zone and migrate toward the root apical meristem by moving in between cells. Although this type of migration creates minimal tissue damage, root‐knot nematode invasion of the root apical meristem induces expression of the ERF109 transcription factor. This eventually promotes tissue regeneration and reduces the inhibitory effect of nematode infection on primary root growth (Zhou *et al*., 2019). Thus, wound signaling can mediate primary root growth compensation in response to damage by stealthily migrating root‐knot nematodes. However, further research is needed to understand whether JA‐dependent wound signaling regulates root architectural changes to compensate for tissue destruction by the more damaging cyst nematodes in the differentiation and mature root zones.

In this study, we hypothesized that local tissue damage by cyst nematode host invasion causes secondary root formation at infection sites via the JA‐dependent ERF109 pathway. By challenging Arabidopsis seedlings with increasing numbers of J2s of the beet cyst nematode *Heterodera schachtii*, we found that secondary root formation is induced at infection sites in a nematode density‐dependent manner. With time course confocal microscopy of JA biosensors and ERF109 reporter lines in Arabidopsis, we provide evidence that secondary root formation is preceded by the transient and local JA‐dependent expression of ERF109. Moreover, the nematode density‐dependent increase in secondary roots is abolished in *coi1‐2* and *erf109* knockout mutants. By selectively applying the auxin biosynthesis chemical inhibitor l‐kynurenine (l‐kyn) to shoots and roots, we further found that the ERF109‐mediated formation of secondary roots is dependent on local auxin biosynthesis. We therefore conclude that tissue damage by host‐invading cyst nematodes induces secondary root formation by altering local auxin biosynthesis via the JA‐dependent ERF109 pathway. Altogether, our results show that damage signaling via the JA‐dependent ERF109 pathway regulates root architectural plasticity in response to cyst nematode infection.

## Materials and Methods

### Plant material and growth conditions

The Arabidopsis (*Arabidopsis thaliana*) lines Col‐0, *pAOS::YFP*_*N*_ (Poncini *et al*., 2017), *DR5::GUS/Col‐0*, and *DR5::GUS/erf109* (Cai *et al*., 2014), *p35S::JAS‐VENUS/p35S::H2B‐RFP* (Larrieu *et al*., 2015), and *pERF109::GFP/Col‐0* (Zhou *et al*., 2019) were used. The *erf109* mutant was chosen because of extensive characterization in previous research (Cai *et al*., 2014; Kong *et al*., 2018; Zhang *et al*., 2019; Ye *et al*., 2020). The weak allele *coi1‐2* mutant (Xu *et al*., 2002) was used since it allows for propagation of homozygous plants and therefore does not need preselection with MeJA, which could interfere with the ERF109 pathway. *pERF109::GFP/coi1‐2* was obtained through crossing followed by the selection of homozygous plants on selective ½ Murashige & Skoog medium containing 15 μg ml^−1^ hygromycin B (Melford Laboratories Ltd, Ipswich, UK) and 20 μg ml^−1^ MeJA (Sigma‐Aldrich). Arabidopsis plants were vertically grown in sterile conditions on modified Knop medium (Sijmons *et al*., 1991) in a growth chamber with a 16 h : 8 h, light : dark photoperiod at 21°C.

### Nematode sterilization

*Heterodera schachtii* (Woensdrecht population from IRS, the Netherlands) cysts were extracted from sand of *Brassica oleracea* infected plants as previously described (Baum *et al*., 2000) and incubated for 7 d in a solution containing 1.5 mg ml^−1^ gentamycin sulfate, 0.05 mg ml^−1^ nystatin, and 3 mM ZnCl_2_. Hatched J2s were purified by centrifugation on a 35% sucrose gradient and surface sterilized for 15 min in a solution containing 0.16 mM HgCl_2_, 0.49 mM NaN_3_, and 0.002% Triton X‐100. After washing three times with sterile tap water, *H. schachtii* J2s were resuspended in a sterile 0.7% Gelrite (Duchefa Biochemie, Haarlem, the Netherlands) solution. A similar concentration of Gelrite solution was used as mock treatment.

### Inoculation density–response curve

Individual Arabidopsis seeds were sown in square Petri dishes. Nine‐day‐old seedlings were inoculated with 0 (mock), 50, 100, 200, 350, or 500 *H. schachtii* J2s. Specifically, two 5 μl drops of solution (with J2s or mock) were pipetted at opposite sides of each seedling while keeping the Petri dishes vertical. This allowed for a homogeneous smear of J2s along the whole length of the root. At 7 d post‐inoculation (dpi), scans were made of whole seedlings using an Epson Perfection V800 photo scanner (Epson, Nagano, Japan). The root architecture (total root length, primary root length, and total secondary root length) was measured using the WinRhizo package for Arabidopsis (Regent Instruments Inc., Québec, Canada). For the *coi1‐2* mutant, primary root length was measured manually because of the convoluted root system. The number of root tips was counted manually based on the scans. Furthermore, nematodes within the roots were stained with acid fuchsin and counted as previously described (Warmerdam *et al*., 2018). For comparisons between genotypes, the background effect of the mutation on the root architecture was corrected by normalizing each measured component in infected seedlings to the average respective component in mock‐inoculated roots. Additionally, the presence of clusters and the number of secondary roots per cluster were scored using a binocular.

### Histology and microscopy

Four‐day‐old Arabidopsis seedlings were inoculated with either 15 *H. schachtii* J2s or mock solution. The choice of using younger seedlings as previously done by Zhou *et al*. (2019) was made to reduce the damage inflicted to the seedling during sample preparation for microscopy. Root architecture was inspected using an Olympus SZX10 binocular with a ×1.5 objective and ×2.5 magnification (Olympus, Tokyo, Japan). Pictures were taken with an AxioCam MRc5 camera (Zeiss). For confocal and brightfield microscopy, single‐nematode infection sites were selected for observation. For histochemical staining of β‐glucuronidase (GUS) activity, seedlings were incubated in a GUS staining solution as previously described (Zhou *et al*., 2019) for 4 h. Stained seedlings were mounted in a chloral hydrate clearing solution (12 M chloral hydrate; 25% glycerol) and inspected with an Axio Imager.M2 light microscope (Zeiss) via a ×20 objective. Differential interference contrast images were taken with an AxioCam MRc5 camera (Zeiss). β‐Glucuronidase saturation was quantified as previously described (Beziat *et al*., 2017) using Fiji software (Schindelin *et al*., 2012). For confocal laser scanning microscopy, seedlings were mounted either in water or in 10 μg ml^−1^ propidium iodide and imaged using a Zeiss LSM 710 system via ×10 and ×40 objectives. The wavelengths used were as follows: 600–640 nm for PI, 500–540 nm for GFP, 520–560 nm for YFP, and 590–680 nm for RFP. For *pAOS::YFP*_*N*_ and *JAS9‐VENUS* reporters, the fluorescent signal was imaged at the focal plane displaying the xylem vessels, where the nematode head is found. For the *pERF109::GFP* reporter, Z‐stacks of six 13 μm slices were made of the entire root depth. Images were taken using Zen 2009 software (Zeiss) and processed using the Fiji software. To make the fluorescence more visible, the brightness was enhanced for all the representative pictures in the same way using Adobe Photoshop 2021. Fluorescence intensity was quantified using the Fiji software. Specifically, the region of interest was selected using a set threshold, and then the integrated density was measured. Z‐stacks were projected using the maximum intensity method.

### Auxin biosynthesis inhibition

For split plate assays, we used the method described by Matosevich *et al*. (2020). For the l‐kyn split plate assay, the four treatment combinations prepared were as follows: MM (modified Knop medium and 0.02% DMSO), KK (modified Knop medium, 10 μM l‐kynurenine (Sigma‐Aldrich), and 0.02% DMSO), MK (l‐kyn only in the root), and KM (l‐kyn only in the shoot). The Yucasin split plate assay is described in Supporting Information Fig. S1. Four‐day‐old Arabidopsis seedlings were inoculated with 15 *H. schachtii* J2s. Sixteen‐hours post‐inoculation (hpi), when J2s are still migrating through the root, seedlings were transferred to the treatment plates, so that the shoot and the hypocotyl were in contact with the medium in the upper half of the plate and the nematode‐infected root was on the medium in the lower half of the plate. For microscopy, seedlings were collected at 3 dpi, and GUS staining was performed. For root architecture inspection, scans were made of whole seedlings at 7 dpi using an Epson Perfection V800 photo scanner. The total number of secondary roots per plant was counted based on the scans. Additionally, the presence of clusters and the number of secondary roots per cluster were scored using an Olympus SZX10 binocular.

### Reverse transcription‐quantitative real‐time PCR

For reverse transcription‐quantitative real‐time PCR (RT‐qPCR) analysis, several hundred root segments (*c*. 0.2 cm) harboring nematode infection sites or similar root segments of mock‐inoculated 12‐d‐old seedlings of Arabidopsis were collected at 12 hpi. Attention was paid not to include root tips and secondary root primordia. Subsequently, RNA extraction and qPCR were performed as previously described (Chopra *et al*., 2021; Hasan *et al*., 2022). *ERF109* was amplified using the primers CTTATGATCGAGCCGCGATT and TCCTCCGTTCCATTGCTCTG (Cai *et al*., 2014; Zhou *et al*., 2019). Three independent biological replicates of the experiment were performed, with three technical replicates per each biological replicate. Relative expression of *ERF109* was calculated based on the endogenous control *18 S rRNA* (Pfaffl, 2001). The average *ERF109* expression in the mock‐inoculated wild‐type roots of the first biological replicate was used as a reference to normalize the average expression in the other samples (Hasan *et al*., 2022).

### Statistical analyses

Statistical analyses were performed using the R software v.3.6.3. The correlation between variables was calculated using Spearman rank‐order correlation coefficient. Significance of the differences between samples was calculated as indicated in the figure legends. The confidence interval of the inoculum density–response curves was calculated by loess regression (as per default in geom_smooth) in R.

## Results

### *Heterodera schachtii* infection induces local formation of secondary roots in a nematode density‐dependent manner

**Fig. 1:**
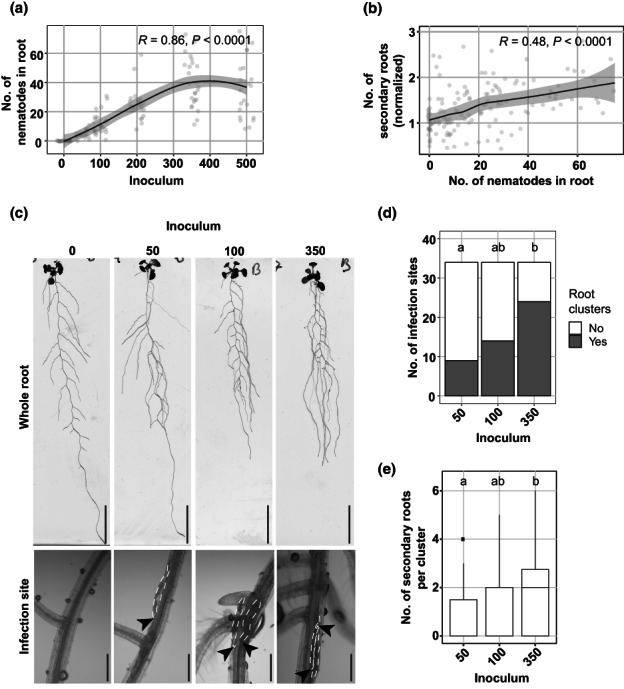
Secondary roots form locally at *Heterodera schachtii* infection sites in a nematode density‐dependent manner. Nine‐day‐old Arabidopsis Col‐0 seedlings were inoculated with increasing numbers of *H. schachtii* second‐stage juveniles (J2s), ranging from 0 (mock) to 500 J2s per seedling. At 7 d post‐inoculation (dpi), scans were made of the root systems, and the total number of secondary roots per plant was counted. Fuchsin staining was performed to count the number of J2s that had penetrated the roots. Additionally, the presence of clusters and the number of secondary roots per cluster were scored. (a) Number of nematodes that successfully penetrated the roots per inoculum. (b) Number of secondary roots formed per number of nematodes inside the roots. The total number of secondary roots in infected seedlings was normalized to the average respective component in uninfected roots and correlated with the number of nematodes inside the roots. Data from three independent biological repeats of the experiment were combined. Correlation (*R*) between two variables was calculated using Spearman's rank‐order correlation coefficient (*n* = 30; *P* < 0.0001). Gray area indicates 95% confidence interval. (c) Representative pictures of whole roots and infection sites in Col‐0 seedlings inoculated with 0 (mock), 50, 100, and 350 J2s. Bars in whole root and infection site pictures are 2 cm and 200 μm, respectively. Black arrowheads indicate the nematode head; white dotted lines outline the nematode body. (d) Proportions of secondary root clusters close to infection sites in Arabidopsis seedlings inoculated with 50, 100, and 350 J2s. Statistical significance was calculated by a pairwise *Z*‐test (*n* = 34; *P* < 0.05). (e) Number of secondary roots within each root cluster in Arabidopsis seedlings inoculated with 50, 100, and 350 J2s. Statistical significance was calculated by pairwise Wilcoxon test followed by false discovery rate correction for multiple comparisons (*n* = 34; *P* < 0.001). For boxplots, the horizontal line represents the median, the whiskers indicate the maximum/minimum range, and the black dots represent the outliers. Different letters indicate statistically different groups.

To test whether tissue damage by invading nematodes in roots triggers the formation of secondary roots, we analyzed root branching upon penetration by increasing numbers of nematodes. We inoculated seedlings with 0, 50, 100, 200, 350, or 500 J2s of *H. schachtii* and counted both the number of nematodes that penetrated the roots and the total number of secondary roots at 7 dpi (Fig. 1). Here, the total number of secondary roots in infected seedlings was normalized to the average respective number in uninfected roots. We found that the number of nematodes that penetrated the roots increased by inoculum density for up to 350 J2s per plant, whereafter it remained the same (Fig. 1a). Furthermore, we observed that the number of penetrated nematodes correlated positively with the total number of secondary roots per plant (Fig. 1b). Next, we investigated whether the clustering of secondary roots around nematode infections sites also correlates with the inoculum density (Fig. 1c,d). For this, we challenged Arabidopsis with four inoculum densities (0, 50, 100, and 350) to establish an incremental increase in the number of nematode infection sites per plant. Infection sites were identified by the local discoloration of root tissue due to cell necrosis along the migratory tract of the nematode (Grundler *et al*., 1994). Roots were counted as clusters when more than one secondary root emerged in the proximity of an infection site. Also, we counted the number of secondary roots per cluster. We found that uninfected seedlings showed a typical pattern of lateral roots regularly distributed along the primary root (Fig. 1c). However, in infected seedlings, clusters of secondary roots emerged close to nematode infection sites in an inoculum density‐dependent manner (Fig. 1c,d). Interestingly, the number of secondary roots per cluster also significantly increased at inoculum density 350 compared with 50 (Fig. 1c,e). Moreover, higher inoculation densities caused more extensive discoloration at the infection sites indicating higher levels of tissue damage. Altogether, these observations showed that infection by *H. schachtii* triggers local density‐dependent formation of secondary roots.

### *Heterodera schachtii* host invasion induces JA biosynthesis and signaling

**Fig. 2:**
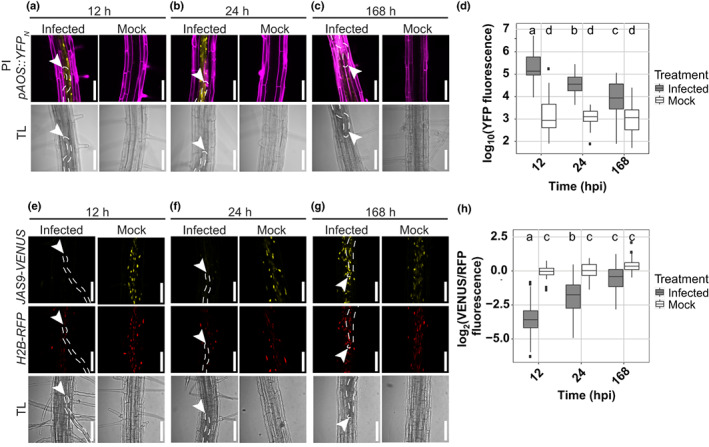
Transient induction of jasmonate (JA) biosynthesis and signaling at *Heterodera schachtii* infection sites. Four‐day‐old Arabidopsis seedlings were either inoculated with 15 *H. schachtii* second‐stage juveniles (J2s) or mock‐inoculated. At 12, 24, and 168 h post‐inoculation (hpi), seedlings were mounted in 10 μg ml^−1^ propidium iodide and then imaged using a fluorescent confocal microscope. Single‐nematode infection sites were selected for observation. (a–c) Representative pictures of infected and noninfected roots expressing the JA biosynthesis marker *pAOS::YFP*_*N*_. To make the fluorescence more visible, the brightness was enhanced for all the representative pictures in the same way. (d) Quantification of YFP intensity in the *pAOS::YFP*_*N*_ line. Values represent the log_10_ of the YFP integrated density. (e–g) Representative pictures of infected and noninfected roots expressing the JA biosensor *p35S::JAS9‐VENUS/p35S::H2B‐RFP*. To make the fluorescence more visible, the brightness was enhanced for all the representative pictures in the same way. (h) Quantification of the JA signaling repressor motif *JAS9*. Values represent log_2_ of the fluorescence ratio between *JAS9‐VENUS* and *H2B‐RFP* raw integrated densities. Data from three independent biological repeats of the experiment were combined. Significance of differences between fluorescent intensities in nematode‐infected and noninfected seedlings over the different time points was calculated by analysis of variance followed by Tukey's HSD test for multiple comparisons (*n* = 30; *P* < 0.0001). For boxplots, the horizontal line represents the median, the whiskers indicate the maximum/minimum range, and the black dots represent the outliers. Different letters indicate statistically different groups. White arrowheads indicate the nematode head; white dotted lines outline the nematode body. TL, transmission light. Bar, 100 μm.

Artificially induced tissue damage can trigger the formation of roots via JA‐dependent signaling pathways. For instance, wounding induces JA‐dependent *de novo* root organogenesis in leaf explants (Zhang *et al*., 2019). Infective juveniles of *H. schachtii* invade the host by destructive thrusts of the oral stylet and release of plant cell wall degrading enzymes causing extensive cell damage during host invasion (Grundler *et al*., 1994; Tytgat *et al*., 2002; Vanholme *et al*., 2007). We hypothesized that secondary root formation in the proximity of *H. schachtii* infection sites might be regulated by JA, in response to tissue damage associated with nematode host invasion. To test our hypothesis, we investigated whether JA biosynthesis and signaling were activated during *H. schachtii* infection using the JA biosynthesis reporter line *pAOS::YFP*_*N*_ (Poncini *et al*., 2017) and the JA signaling biosensor *JAS9‐VENUS* (*p35S::JAS9‐VENUS/p35S::H2B‐RFP*) (Larrieu *et al*., 2015) (Fig. 2). We chose three time points that reflect the early parasitic stages of intracellular host invasion (12 hpi), permanent feeding site initiation (24 hpi), and permanent feeding site expansion (168 hpi) (Tytgat *et al*., 2002; Hewezi *et al*., 2014; Kammerhofer *et al*., 2015; Marhavy *et al*., 2019). Importantly, to avoid interference of signals due to the presence of multiple nematodes at one infection site, we selected single‐nematode infection sites for our observations. We found that infection with *H. schachtii* significantly induces transient expression of *pAOS::YFP*_*N*_, with the highest level of expression at 12 hpi (Fig. 2a–d). Likewise, *JAS9‐VENUS* showed a strong JA signaling activity (i.e. low VENUS : RFP ratio) in infected roots at 12 hpi, which decreased over time to the level of uninfected root tissue at 168 hpi (Fig. 2e–h). These observations demonstrated that both JA biosynthesis and JA signaling are strongly induced during and shortly after *H. schachtii* host invasion close to the nematode infection site. We therefore concluded that tissue damage caused by *H. schachtii* during intracellular host invasion triggers local JA biosynthesis and signaling in Arabidopsis.

### COI1‐mediated JA signaling regulates *ERF109* expression upon *H. schachtii* infection

**Fig. 3:**
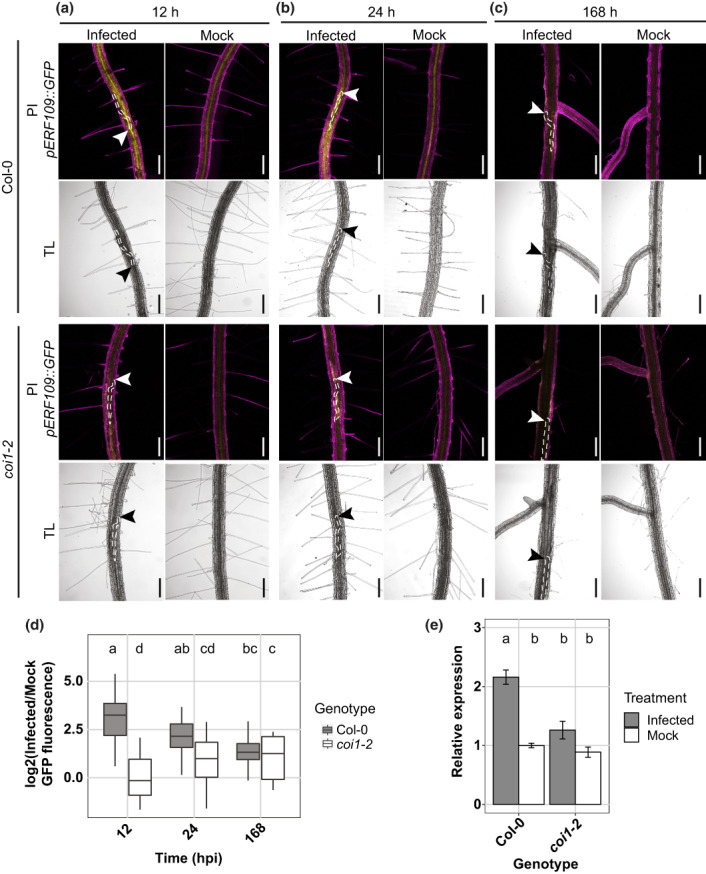
*ERF109* expression upon *Heterodera schachtii* host invasion is dependent on COI1‐mediated jasmonate signaling. (a–d) Four‐day‐old Arabidopsis seedlings were either inoculated with 15 *H. schachtii* second‐stage juveniles (J2s) or mock‐inoculated. At 12, 24, and 168 h post‐inoculation (hpi), seedlings were mounted in 10 μg ml^−1^ propidium iodide (PI) and then imaged using a fluorescent confocal microscope. Single‐nematode infection sites were selected for observation. (a–c) Representative pictures of infected and mock‐inoculated seedlings expressing the *pERF109::GFP* construct in either wild‐type Col‐0 or mutant *coi1‐2* background at 12 hpi (a), 24 hpi (b), and 168 hpi (c). To make the fluorescence more visible, the brightness was enhanced for all the representative pictures in the same way. (d) Quantification of *pERF109::GFP* fluorescent intensity induced by infection of Col‐0 and *coi1‐2* roots. Values represent log_2_ of the fluorescence ratio between the GFP integrated density of infected and noninfected roots. Data from two independent biological repeats of the experiment were combined. Significance of differences between fluorescent intensities in Col‐0 and *coi1‐2* roots over the different timepoints was calculated by analysis of variance followed by Tukey's HSD test for multiple comparisons (*n* = 20; *P* < 0.05). For boxplots, the horizontal line represents the median and the whiskers indicate the maximum/minimum range. Different letters indicate statistically different groups. White and black arrowheads indicate the nematode head; white dotted lines outline the nematode body. TL, transmission light. Bar, 200 μm. (e) Twelve‐day‐old Col‐0 and *coi1‐2* Arabidopsis plants were inoculated with *H. schachtii*. At 12 hpi, RNA was extracted from root segments of *c*. 0.2 cm harboring nematode infection sites or similar root segments of mock‐inoculated seedlings. Data represent three independent biological replicates with three technical replicates per biological replicate. Relative expression of *ERF109* was first calculated based on the endogenous control *18 s rRNA* and then normalized to the mock‐inoculated wild‐type samples in the first biological replicate. Significance of differences between *ERF109* relative expression in Col‐0 and *coi1‐2* infected roots was calculated by ANOVA followed by Tukey's HSD test for multiple comparisons (*n* = 3 biological replicates; *P* < 0.01). Different letters indicate statistically different groups. Error bars represent SE of the mean.

Root tip resection or wounding in leaf explants induces *ERF109* expression in a COI1‐dependent manner (Zhang *et al*., 2019; Zhou *et al*., 2019). To determine whether *H. schachtii‐*induced JA signaling also triggers *ERF109* expression, we monitored *pERF109::GFP* expression within single‐nematode infection sites in the *coi1‐2* mutant and wild‐type Arabidopsis Col‐0 plants during the early stages of infection by *H. schachtii* (Fig. 3). Similar to that observed for JA biosynthesis and signaling, *ERF109* expression was induced at early time points (12 and 24 hpi) of *H. schachtii* infection around the migratory track of the nematodes in wild‐type Col‐0 (Fig. 3a–c). Moreover, in the *coi1‐2* mutant, *pERF109::GFP* fluorescence was significantly reduced compared with wild‐type Arabidopsis (Fig. 3d). Nevertheless, we observed a slight increase in fluorescence in *coi1‐2* mutant over time, which reached the fluorescence levels detected in Col‐0 at 168 hpi. The fluorescence detected in wild‐type Col‐0 and *coi1‐2* mutant at 168 hpi might be caused by tissue autofluorescence from the cell walls of the permanent feeding sites (Hoth *et al*., 2005). Since *pERF109::GFP* has a nuclear‐cytoplasmic localization (Zhou *et al*., 2019) due to GFP diffusion into the nucleus (Hanson & Kohler, 2001), autofluorescence from cell walls in syncytia cannot be easily distinguished from the cytoplasmic part of the *pERF109::GFP* signal. However, after quantifying only nuclear‐localized *pERF109::GFP*, the initially observed fluorescence in the nematode‐infected wild‐type Col‐0 and *coi1‐2* mutant plants at 168 hpi was not detected anymore, pointing at autofluorescence as the most plausible cause (Fig. S2). To independently verify a COI1‐dependent regulation of *ERF109* expression, we also performed a RT‐qPCR on ERF109 transcripts in root segments containing nematode infection sites collected from *coi1‐2* and wild‐type Col‐0 at 12 hpi. Consistent with the observed regulation of *pERF109::GFP* fluorescence, we found significantly fewer transcripts of *ERF109* in *coi1‐2* than in wild‐type Col‐0 (Fig. 3e). We therefore concluded that *H. schachtii* induces *ERF109* expression during host invasion in a JA‐dependent manner.

### COI1 and ERF109 regulate secondary root formation upon *H. schachtii* infection

**Fig. 4:**
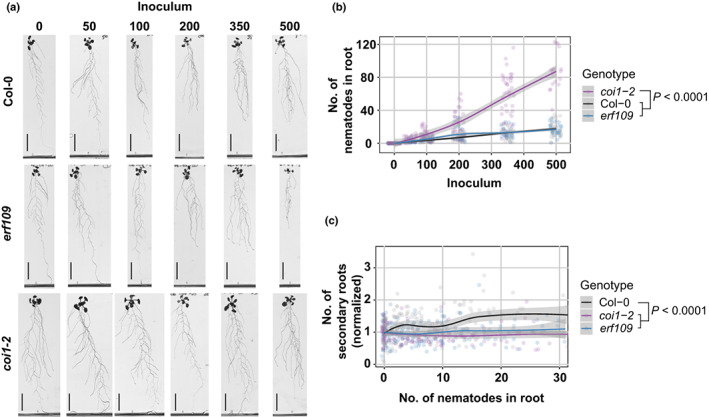
ERF109 and COI1 regulate the nematode density‐dependent secondary root formation that is triggered by *Heterodera schachtii* infections. Nine‐day‐old wild‐type Col‐0, *erf109*, and *coi1‐2* seedlings were inoculated with increasing numbers of *H. schachtii* second‐stage juveniles (J2s), ranging from 0 (mock) to 500 J2s per seedling. At 7 d post‐inoculation (dpi), scans were made of the root systems, and the number of secondary roots per plant was counted. Fuchsin staining was performed to count the number of J2s that had penetrated the roots. (a) Representative pictures of wild‐type Col‐0, *erf109*, and *coi1‐2* infected seedlings at 7 dpi. (b) Number of nematodes that successfully penetrated the roots per inoculum. (c) Secondary roots formed per number of nematodes inside the roots. The total number of secondary roots in infected seedlings was normalized to the average respective component in mock‐treated roots. Data from three independent biological repeats of the experiment were combined. Significance of differences between genotypes was calculated by analysis of variance followed by Tukey's HSD test for multiple comparisons (*n* = 30; *P* < 0.0001). Gray area indicates 95% confidence interval. Bar, 2 cm.

Next, we asked whether the activation of JA‐dependent expression of *ERF109* is required for the formation of secondary roots during *H. schachtii* infections. If this holds true, the nematode density‐dependent increase in secondary roots observed for wild‐type Col‐0 should be altered in both *coi1‐2* and *erf109* mutants. To test this, we performed the same density–response experiment as shown in Fig. 1(a,b). At 7 dpi, the number of nematodes that had successfully penetrated the roots did not differ significantly between wild‐type Col‐0 and the *erf109* mutant (Fig. 4). By contrast, the number of nematodes was significantly higher in roots of the *coi1‐2* mutant than wild‐type Arabidopsis plants, indicating a role of COI1 in plant susceptibility to penetration by *H. schachtii* (Fig. 4b). However, it must be noted that the uninfected *coi1‐2* mutant had a much larger root system than wild‐type Arabidopsis Col‐0 (Fig. S3), which also may influence the number of nematode penetrations. Nevertheless, while nematode infections in wild‐type Arabidopsis induced the formation of secondary roots, no such increase was observed for *erf109* and *coi1‐2* mutants (Fig. 4c). In conclusion, both COI1 and ERF109 regulate the density‐dependent induction of secondary root formation by *H. schachtii*. This induction of secondary root formation is independent from plant susceptibility to nematode penetration.

### ERF109‐mediated induction of secondary root formation compensates for primary root growth inhibition by *H. schachtii*

**Fig. 5:**
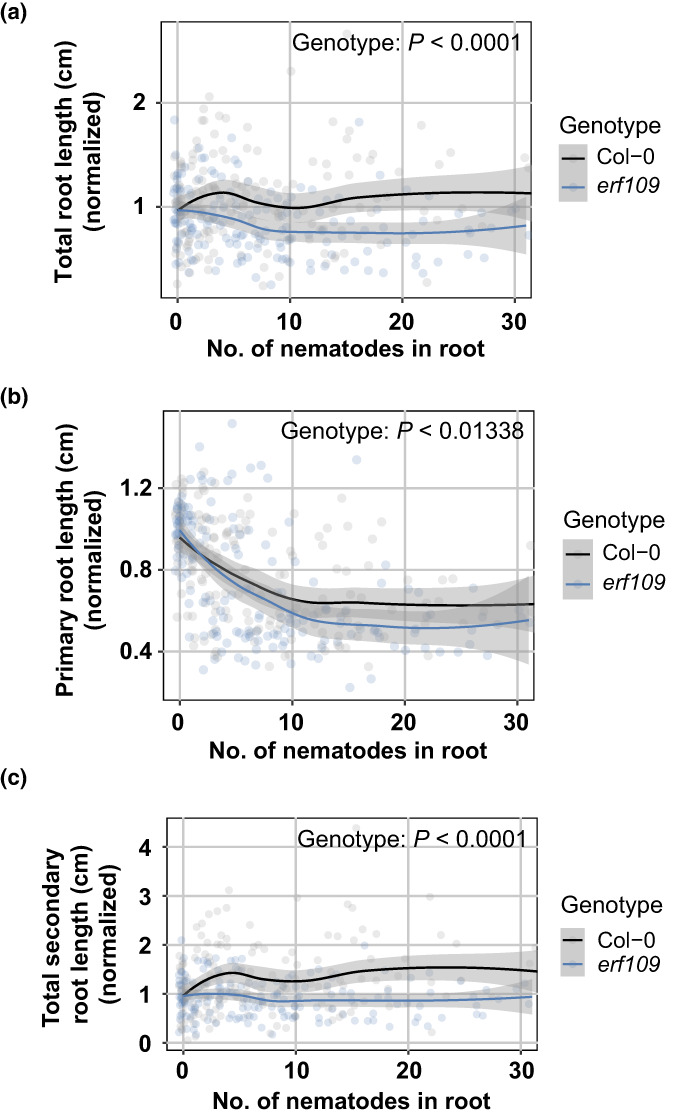
ERF109‐mediated secondary root formation allows for maintenance of total root length despite primary root growth inhibition by *Heterodera schachtii*. Nine‐day‐old Col‐0 and *erf109* Arabidopsis seedlings were inoculated with increasing *H. schachtii* densities ranging from 0 (mock) to 500 second‐stage juveniles (J2s) per seedling. At 7 d post‐inoculation, scans were made of the root systems, and the root length was measured using WinRhizo. Total, primary, and secondary root length were normalized to the average respective component in mock‐treated roots. Fuchsin staining was performed for counting the number of J2s that penetrated the roots. (a) Total root length per number of nematodes in the roots. (b) Primary root length per number of nematodes in the roots. (c) Total secondary root length per number of nematodes in the roots. Data from three independent biological repeats of the experiment were combined. Significance of differences between genotypes was calculated by analysis of variance (*n* = 30). Gray area indicates 95% confidence interval.

The induction of secondary root formation by cyst nematodes might compensate for a possible inhibition of root growth by nematode invasion. To test this hypothesis, we investigated whether the total length of the entire root system, the primary root length, and the total length of the secondary roots were altered in the infected *erf109* mutant compared with wild‐type Col‐0 (Fig. 5). To eliminate the background effect of the mutation on the root architecture, we normalized each measured component in infected seedlings to the average respective component in uninfected roots. We found that the total length of the root system of wild‐type Col‐0 at increasing numbers of nematodes remains similar to that of uninfected plants (i.e. close to 1 in Fig. 5a). By contrast, the total length of the entire root system in the *erf109* mutant decreased by nematode density as compared to uninfected plants. As the total length of the root system is the sum of the lengths of the primary roots and the secondary roots, we also analyzed these components separately. The primary root length of both wild‐type Col‐0 and the *erf109* mutant declined by nematode density (Fig. 5b). This decline was slightly but significantly exacerbated by the *erf109* mutation. However, we found a more striking difference in the total length of the secondary roots between wild‐type Col‐0 and the *erf109* mutant (Fig. 5c). In wild‐type Col‐0, we observed a significant increase in the total length of the secondary roots by nematode density, sufficient to compensate for the loss in primary root length. However, we observed no significant increase in the total length of the secondary roots by nematode density in the *erf109* mutant, which explains why the total length of the root system by nematode density remained stable for wild‐type Col‐0, but not for the *erf109* mutant. Based on our data, we conclude that ERF109‐mediated formation of secondary roots compensates for primary root growth inhibition by *H. schachtii*.

### ERF109 regulates local auxin biosynthesis at the nematode infection site

**Fig. 6:**
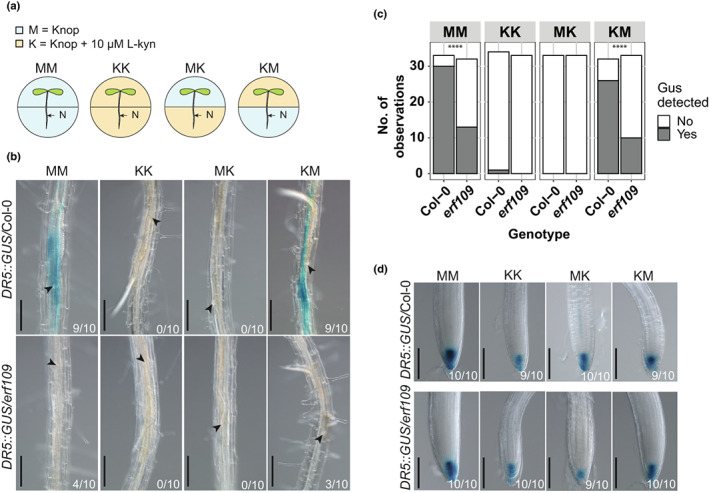
ERF109 regulates local auxin biosynthesis at *Heterodera schachtii* infection sites. Four‐day‐old Arabidopsis Col‐0 and *erf109* seedlings expressing the auxin *DR5::GUS* reporter were infected with 15 *H. schachtii* second‐stage juveniles (J2s). At 16 h post‐inoculation, seedlings were transferred to treatment plates. Four treatment combinations were prepared as follows: MM (modified Knop medium and 0.02% DMSO), KK (modified Knop medium, 10 μM l‐kyn and 0.02% DMSO), MK (l‐kyn only in the root), KM (l‐kyn only in the shoot). At 3 d post‐inoculation, GUS staining assay was performed for 4 h, and seedlings were imaged. Single‐nematode infection sites were selected for observation. (a) Experimental design with Arabidopsis seedlings transferred to split plates with modified Knop medium either with or without l‐kyn. N, nematode. (b) *DR5::GUS* expression at nematode infection sites in wild‐type Col‐0 and *erf109* roots in the four different treatment combinations with or without l‐kyn applied to shoots and/or roots. (c) Number of observations with (Yes) or without (No) GUS staining at the nematode infection sites in roots of wild‐type Col‐0 and *erf109* plants. Statistical significance was calculated by a pairwise *Z*‐test (*n* = 33; ****, *P* < 0.0001). (d) *DR5::GUS* expression in the root tips of Col‐0 and *erf109* roots. Black arrowheads indicate the nematode head. Frequencies at the bottom right corner indicate how many times GUS staining was observed in one of the three independent biological repeats of the experiment. Bar, 200 μm.

ERF109 mediates JA‐induced secondary root formation by directly binding to the promoter of auxin biosynthesis genes *ASA1* and *YUC2* (Cai *et al*., 2014). We hypothesized that ERF109 regulates secondary root formation by inducing local auxin biosynthesis at the nematode infection site. Thus, we used a split plate assay containing growth media with and without l‐kyn to chemically inhibit auxin biosynthesis in the shoots and/or the roots of infected wild‐type and *erf109* plants (Fig. 6). The local accumulation of auxin was monitored using the *DR5::GUS* reporter (Fig. 6a). When seedlings were grown on regular medium or when auxin biosynthesis was inhibited by l‐kyn only in the shoots, *DR5::GUS* was expressed at nematode infection sites in wild‐type Col‐0 seedlings. However, when auxin biosynthesis was inhibited in both shoots and roots or only in the roots by treatment with l‐kyn, no *DR5::GUS* expression was observed (Fig. 6b,c). This suggested that auxin accumulation at nematode infection sites was dependent on local auxin biosynthesis in the roots. Importantly, we observed that the auxin accumulation at nematode infection sites via root‐localized auxin biosynthesis was disrupted in the *erf109* mutant. Indeed, *DR5::GUS* expression was significantly lower at the nematode infection sites in *erf109* seedlings than wild‐type Col‐0 when auxin biosynthesis was permitted in the root (Fig. 6b,c). To determine whether the differences in *DR5::GUS* between the two Arabidopsis genotypes were only local at the nematode infection site or systemic throughout the root system, we also looked at *DR5::GUS* expression in root tips (Figs 6d, S4). In contrast to nematode infection sites, when auxin biosynthesis was inhibited only in the shoots, we observed no difference between *erf109* and wild‐type Col‐0 in *DR5::GUS* expression in the root tip (Figs 6d, S4). Since l‐kyn has been shown to also inhibit ethylene‐induced auxin biosynthesis (He *et al*., 2011), we also performed the experiment using the auxin biosynthesis inhibitor Yucasin (Yuc). Due to the higher concentration of DMSO used to dissolve Yuc, an overall lower frequency of *DR5:GUS* staining was observed. Nevertheless, the Yuc split plate assay showed the same trend as the l‐kyn experiment (Fig. S1). From these results, we concluded that ERF109 regulates local auxin biosynthesis at infection sites of *H. schachtii*.

### ERF109‐induced secondary root formation upon *H. schachtii* infection is dependent on local auxin biosynthesis

**Fig. 7:**
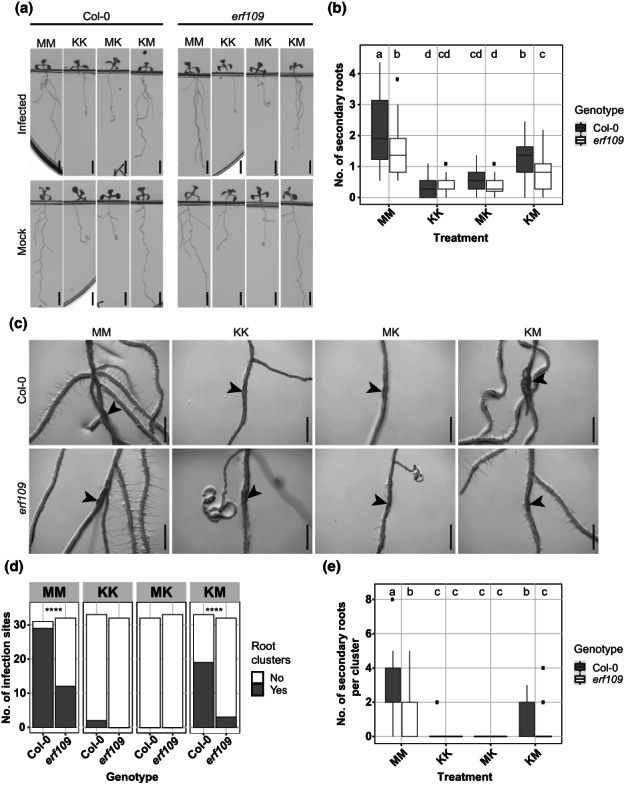
ERF109‐dependent local auxin biosynthesis regulates secondary root formation upon *Heterodera schachtii* infection. Four‐day‐old Arabidopsis Col‐0 and *erf109* seedlings were either infected with 15 *H. schachtii* second‐stage juveniles (J2s) or mock‐inoculated. At 16 h post‐inoculation, seedlings were transferred to treatment plates. Four treatment combinations were prepared as follows: MM (modified Knop medium and 0.02% DMSO), KK (modified Knop medium, 10 μM l‐kyn and 0.02% DMSO), MK (l‐kyn only in the root), and KM (l‐kyn only in the shoot). At 7 d post‐inoculation, scans were made of the root systems, and the total number of secondary roots per plant was counted. Additionally, the presence of clusters and the number of secondary roots per cluster were scored. (a) Representative pictures of wild‐type Col‐0 and *erf109* mutant seedlings. (b) Number of secondary roots in infected vs noninfected roots of wild‐type Col‐0 and *erf109* seedlings. Data from two independent biological repeats of the experiment were combined. Significance of differences in secondary roots between the different treatment combinations was calculated by analysis of variance followed by Tukey's HSD test for multiple comparisons (*n* = 43–45; *P* < 0.05). (c) Representative images of nematode infection sites in wild‐type Col‐0 and *erf109* mutant. (d) Number of secondary root clusters that are associated with *H. schachtii* infection sites. Data from two independent biological repeats of the experiment were combined. Statistical significance was calculated by a pairwise *Z*‐test (*n* = 31–33; ****, *P* < 0.0001). (e) Number of secondary roots per cluster. Data from two independent biological repeats of the experiment were combined. Significance of differences between secondary roots within a cluster was calculated by aligned rank transform for nonparametric factorial ANOVA followed by Tukey's HSD test for multiple comparisons (*n* = 31–33; *P* < 0.0001). For boxplots, the horizontal line represents the median, the whiskers indicate the maximum/minimum range, and the black dots represent the outliers. Difference in letters indicates statistically different groups. Black arrowheads indicate the infection site. Bar, 0.5 cm.

We found that ERF109 regulates local auxin biosynthesis at *H. schachtii* infection sites. This raised the question of whether the ERF109‐mediated secondary root formation upon *H. schachtii* infection is dependent on this local biosynthesis of auxin. To test this, we inoculated 4‐d‐old wild‐type Col‐0 and *erf109* seedlings with either 15 *H. schachtii* J2s or a mock solution. At 16 hpi, seedlings were transferred to the four previously described split plates containing medium with and without 10 μM l‐kyn (Fig. 6a). At 7 dpi, the total number of secondary roots was scored. As expected, the different treatment combinations with and without l‐kyn in the shoots and/or roots led to a different number of lateral roots in the uninfected roots (Fig. S5). Therefore, to calculate the number of additional secondary roots induced by nematode infection, the number of secondary roots in infected roots was normalized to the average respective component in uninfected roots. Additionally, we scored how often a cluster of roots occurs in the proximity of an infection site and the number of secondary roots per cluster (Fig. 7). When auxin biosynthesis was inhibited in both shoots and roots or only in the roots, no additional secondary roots formed in infected Col‐0 wild‐type seedlings (Fig. 7a,b). Consistently, no clusters of secondary roots were found at nematode infection sites (Fig. 7c–e). However, inhibition of auxin biosynthesis in the shoots alone led to a significant reduction in the total number of secondary roots in infected seedlings (Fig. 7a,b) as well as in the number of clusters and the number of secondary roots per cluster compared with when auxin biosynthesis was permitted in both shoots and roots (Fig. 7c–e; treatment MM vs KM). Thus, secondary root formation upon *H. schachtii* infection is dependent on local auxin biosynthesis, although polar auxin transport from the shoots might still play a role. Furthermore, the mutation in *erf109* strongly affected secondary root formation when auxin biosynthesis was permitted in the roots. Indeed, a significant decrease in the number of additional secondary roots, the number of clusters of secondary roots, and the number of secondary roots per cluster was observed for *erf109* than wild‐type Col‐0 (Fig. 7). Altogether, we concluded that ERF109‐dependent secondary root formation upon *H. schachtii* infection relies at least partially on local auxin biosynthesis.

## Discussion

Root architecture plasticity in response to stress by soil‐borne pathogens and pests is a largely unexplored field of research. Root parasitism by cyst nematodes is often associated with the formation of secondary roots in the proximity of infection sites (Grymaszewska & Golinowski, 1991; Goverse *et al*., 2000; Lee *et al*., 2011). However, the molecular mechanisms regulating secondary root formation in response to cyst nematode infection have thus far remained unclear. Here, we provide evidence for a model wherein formation of secondary roots near *H. schachtii* infection sites is triggered by tissue damage caused by nematode invasion. This response is regulated by the JA‐dependent ERF109‐activated local biosynthesis of auxin.

Our data demonstrate that secondary root formation is most likely initiated by tissue damage brought about by cyst nematode infections. The number of secondary roots induced by *H. schachtii* correlated positively with the number of nematodes that penetrated the roots. This increase in the number of secondary roots may be simply due to an increase in the number of infection sites. However, we also observed more nematodes within infection sites at higher inoculation densities, which correlated well with the number of secondary roots per infection site. This may mean that infection sites containing multiple nematodes developed a higher number of secondary roots per cluster than single‐nematode‐associated infection sites. Moreover, we saw more extensive root tissue damage (i.e. root discoloring) at infection sites harboring multiple nematodes. We therefore consider tissue damage by infective juveniles inside roots as the likely cause of enhanced local secondary root formation.

Tissue damage in Arabidopsis leaf explants triggers *de novo* root organogenesis in a JA‐dependent manner (Zhang *et al*., 2019). We found that intracellular host invasion by *H. schachtii* transiently induces JA biosynthesis and signaling and that the JA receptor mutant *coi1‐2* is defective in secondary root formation upon *H. schachtii* infection. Our results are in line with whole transcriptome analyses of root segments of Arabidopsis harboring migrating juveniles of *H. schachtii* at 10 hpi, which also showed that JA biosynthesis and signaling genes are upregulated during host invasion (Kammerhofer *et al*., 2015; Mendy *et al*., 2017). By contrast, recent reports indicate that host invasion by *H. schachtii* does not activate the JA signaling biosensor *JAZ10::NLS‐3xVENUS* in Arabidopsis roots (Marhavy *et al*., 2019). The discrepancy between our observations with the *JAS9‐VENUS* biosensor and the observations with the *JAZ10::NLS‐3xVENUS* biosensor might be due to differences in sensitivity of both sensor constructs. Compared to *JAZ10::NLS‐3xVENUS*, the *JAS9‐VENUS* biosensor is particularly sensitive to biologically active JA (JA‐isoleucine), enabling the visualization of local JA signaling in response to stress in Arabidopsis roots at a high spatiotemporal resolution (Larrieu *et al*., 2015). Furthermore, *JAS9‐VENUS* has been used to monitor the dynamics of JA signaling in response to single‐cell ablation and intercellular migration of the less‐damaging root‐knot nematodes in Arabidopsis roots (Zhou *et al*., 2019). Therefore, based on the activity of the *JAS9‐VENUS* biosensor in our experiments, we conclude that the tissue damage associated with host invasion triggers a JA signal in cells close to the infection site of *H. schachtii*. Moreover, the transient nature of the JA signal suggests that the damage trigger decreases after nematode host invasion or that JA signaling is actively suppressed by *H. schachtii* when infective juveniles become sedentary.

Jasmonate signaling during *H. schachtii* migration also results in activation of plant defense responses (Kammerhofer *et al*., 2015). We observed that the *coi1‐2* mutant is more susceptible to penetration by *H. schachtii*, which is in line with previous findings showing a negative effect of exogenous JA on *H. schachtii* penetration rate (Kammerhofer *et al*., 2015). However, after nematode penetration, COI1 does not affect the rate at which J2s induce a permanent feeding site (Marhavy *et al*., 2019). Altogether, these findings suggest that JA signaling both negatively regulates host penetration rate by *H. schachtii* and mediates secondary root formation at *H. schachtii* infection sites.

The damage‐induced formation of secondary roots by *H. schachtii* appears to be regulated by the JA‐dependent expression of *ERF109*. We found that the expression of *ERF109*, which showed the same transient induction pattern as the JA biosynthesis reporter *AOS* and *JAS9‐VENUS* biosensor, was abrogated in the *coi1‐2* mutant. Moreover, the *erf109* mutant was as defective as the *coi1‐2* mutant in the density‐dependent secondary root formation upon *H. schachtii* infection. Consistent with our data, *ERF109* expression showed a COI1‐dependent transient expression upon wounding in leaf explants (Zhang *et al*., 2019). Furthermore, the *erf109* mutation also disrupted the induction of secondary root formation by exogenous application of JA (Cai *et al*., 2014). Altogether, our findings show that tissue damage by invading nematodes triggers a JA signal, which induces the ERF109‐dependent formation of secondary roots.

Next, our data provide evidence that damage‐induced activation of *ERF109* regulates the formation of secondary roots via local auxin biosynthesis. The local accumulation of auxin at nematode infection sites (i.e. expression of the auxin reporter *DR5::GUS*) was strongly reduced in the *erf109* mutant than in wild‐type plants. However, when auxin biosynthesis was blocked in whole seedlings or only in roots, the local accumulation of auxin at nematode infection sites was completely abolished in both the *erf109* mutant and the wild‐type Arabidopsis. Taken together, this demonstrates that auxin accumulation at nematode infection sites is at least partially dependent on ERF109‐regulated local auxin biosynthesis. Importantly, the patterns observed for local accumulation of auxin at nematode infection sites matched the patterns of secondary root formation in the absence or presence of the auxin biosynthesis inhibitor. The inhibition of auxin biosynthesis in the roots, but not in the shoots, abolished the formation of secondary roots upon nematode infection. Previously, ERF109 was shown to regulate secondary root formation by binding the promoter of auxin biosynthesis genes upon exogenous application of JA (Cai *et al*., 2014). Here, our data show that tissue damage by nematodes activates JA signaling and subsequently induces ERF109, which in turn regulates secondary root formation via local biosynthesis of auxin.

After blocking auxin biosynthesis in the shoots, we observed auxin accumulation and formation of secondary roots at nematode infection sites, which indicates that polar auxin transport from the shoots is not required for secondary root formation at nematode infection sites. Nevertheless, we noted a quantitative effect of the inhibition of auxin biosynthesis in shoots, leading to the formation of fewer secondary root clusters and fewer secondary roots per cluster as compared to untreated plants. This implies that polar auxin transport from the shoots may still play a complementary role in secondary root formation at nematode infection sites, albeit below the detection levels of the *DR5::GUS* reporter. Polar auxin transport from the shoots and further redistribution in root tissue results from the coordinated activities of auxin influx and efflux carrier proteins (Petrasek & Friml, 2009). Lee *et al*. (2011) showed that *H. schachtii* induces the formation of secondary roots in double *aux1lax3* and quadruple *aux1lax1lax2lax3* influx carrier mutants, which are otherwise unable to form secondary roots. This suggests that the accumulation of auxin and subsequent formation of secondary roots may be regulated independently of the activity of these influx carriers. There is ample evidence that auxin efflux carriers (i.e. PIN proteins) are important for the susceptibility of Arabidopsis to infections of *H. schachtii* (Grunewald *et al*., 2009). However, if and how they might contribute to the accumulation of auxin underlying the damage‐induced formation of secondary roots needs further investigation.

Here, we demonstrate that ERF109‐mediated local adaptations in root architecture compensate for primary root growth inhibition in response to nematode infection. In wild‐type Arabidopsis, increasing densities of J2s led to a decline in the length of infected primary roots. However, this reduction in the length of infected primary roots did not result in a smaller root system, because of an increase in the total length of secondary roots. Our data show that these adaptations in root architecture depend on the transient and local activation of *ERF109* by JA at nematode infection sites. Consistently, the JA signaling mutant *coi1‐2* showed a similar impairment as *erf109* in compensating primary root length inhibition by an increase in total secondary root length (Fig. S6). Nevertheless, since COI1 also affects plant susceptibility to nematode penetration, more complex defense vs growth trade‐offs may influence root growth in the *coi1‐2* mutant. Importantly, loss‐of‐function mutations in *ERF109* do not alter the susceptibility of Arabidopsis to *H. schachtii* penetration but instead affect root architecture plasticity in response to nematode infection. Further research is needed to understand whether ERF109‐mediated compensatory adaptations in root architecture could mediate tolerance of Arabidopsis to infections by *H. schachtii*.

It was previously shown that meristem damage caused by *M. incognita* root tip penetration triggers regeneration via JA‐ and ERF109‐mediated damage signaling (Zhou *et al*., 2019). Here, we show that *H. schachtii* penetration of the mature root zone causes damage‐induced secondary root formation, which compensates for primary root growth inhibition. Therefore, we consider root tip regeneration and secondary root formation as two different outcomes of the same compensatory mechanism in response to tissue damage in different root zones.

Furthermore, we show the first case of a naturally occurring and biotic stress that triggers damage signaling‐mediated secondary root formation. Primary roots can form two types of secondary roots (Sheng *et al*., 2017). One type, referred to as a lateral root, forms during the physiological postembryogenic development of plants and is regulated by ARF7 and ARF19 auxin response factors. The other type is induced by sterile mechanical injury of the mature root zone, soil penetration, or osmotic stress and is dependent on the transcription factor WOX11. Sterile mechanical injury causes a different type of root tissue damage compared with a biotic stress, such as cyst nematodes (Marhavy *et al*., 2019). Sterile mechanical injury damages many root cells at one time. Instead, cyst nematode host invasion causes the rupture of multiple single cells one after the other over the course of many hours (Wyss & Zunke, 1986). Thus, our results provide biological relevance for a mechanism so far only observed upon artificial conditions.

As a natural trigger for damage signaling, *H. schachtii* can be used to further elucidate the pathway leading to secondary root formation. ERF109 was previously found to be responsive to reactive oxygen species (ROS) (Kong *et al*., 2018). It would be interesting to test whether ROS mediates ERF109‐dependent secondary root formation upon *H. schachtii* infection. Furthermore, follow‐up research could investigate whether damage receptors activated during *H. schachtii* migration (Shah *et al*., 2017) act upstream of ERF109. The root‐knot nematode *M. javanica* triggers the expression of LBD16, a downstream target of both WOX11, and ARF7 and ARF19 (Cabrera *et al*., 2014; Olmo *et al*., 2017). Moreover, *M. javanica* infection of primary roots induces secondary root formation independently from ARF7 and ARF19 (Olmo *et al*., 2017). This suggests that nematode‐induced secondary root formation could be regulated by WOX11. However, whether WOX11‐mediated secondary root formation acts downstream of the ERF109‐damage signaling pathway remains unknown.

**Fig. 8:**
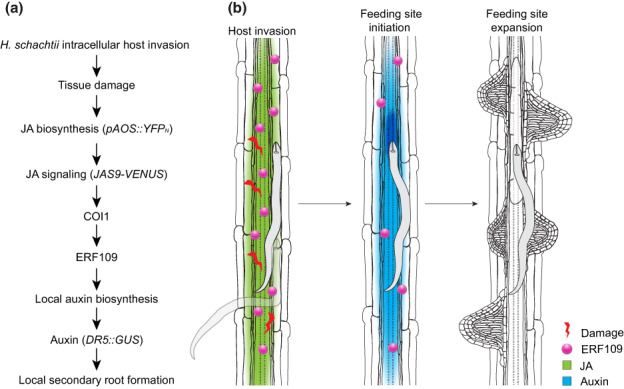
Model of the pathway regulating *Heterodera schachtii*‐induced secondary root formation. (a) Intracellular invasion of host roots by *H. schachtii* causes tissue damage, which triggers jasmonate (JA) biosynthesis (*pAOS::YFP*_*N*_). JA signaling (*JAS9‐VENUS*) via COI1 induces expression of *ERF109*, which leads to auxin accumulation (*DR5::GUS*) via local auxin biosynthesis. ERF109‐mediated local auxin biosynthesis finally results in the formation of secondary roots at *H. schachtii* infection sites. (b) Graphical model illustrating the pathway investigated in this paper.

In summary, we showed that *H. schachtii* triggers the formation of secondary roots via JA‐ and ERF109‐mediated damage signaling (Fig. 8). Furthermore, ERF109‐mediated secondary root formation compensates for primary root growth inhibition associated with *H. schachtii* infection. Thus, damage signaling‐induced formation of secondary roots points at a novel mechanism underlying plant root architecture plasticity to biotic stress. Further research is needed to investigate whether damage‐induced root architecture plasticity can contribute to plant tolerance to belowground herbivory.

## Competing interests

None declared.

## Author contributions

JLL‐T, GS, NG, J‐JW, AG, WZ and VW conceived the project. NG, J‐JW, MSH and LS designed the experiments and performed data collection. WZ and VW provided most of the Arabidopsis mutants and granted access to the confocal microscope. WZ performed the crossing to obtain the *pERF109::GFP/coi1‐2* Arabidopsis line, while homozygous plants were selected by both WZ and NG. Data were analyzed and interpreted by NG, J‐JW, MSH and MGS. NG, JLL‐T, GS and JJW wrote the article with inputs from AG, MGS, VW, WZ, MSH, LS and FMWG. NG and J‐JW contributed equally to this work. GS and JLL‐T contributed equally to this work.

## Supporting information


**Fig. S1** Yucasin split plate assay showing that ERF109 regulates local auxin biosynthesis at the nematode infection site.
**Fig. S2** Induction of *pERF109::GFP* nuclear fluorescence by *Heterodera schachtii* host invasion is disrupted in the *coi1‐2* Arabidopsis mutant.
**Fig. S3** Root architecture of uninfected *coi1‐2* and *erf109* Arabidopsis plants differs from wild‐type Col‐0 plants.
**Fig. S4** *DR5::GUS* expression at the root tip does not differ between infected wild‐type Col‐0 and *erf109* seedlings when auxin biosynthesis is inhibited only in the shoot.
**Fig. S5** Number of lateral roots in noninfected wild‐type Col‐0 and *erf109* mutant seedlings is affected by l‐kyn treatment.
**Fig. S6** COI1‐mediated secondary root formation allows for maintenance of total root length despite primary root growth inhibition by *Heterodera schachtii*.
Please note: Wiley is not responsible for the content or functionality of any Supporting Information supplied by the authors. Any queries (other than missing material) should be directed to the *New Phytologist* Central Office.

## Data Availability

The data that support the findings of this study are available in the Supporting Information of this article.
