# Supplementary material for: Root architecture plasticity in response to endoparasitic cyst nematodes is mediated by damage signaling
Source: New Phytol. 2022 Dec 1;237(3):807–22. doi: 10.1111/nph.18570 (PMC10108316; doi:10.1111/nph.18570)
Supplement: Supplementary file 1 — Fig. S1 Yucasin split plate assay showing that ERF109 regulates local auxin biosynthesis at the nematode infection site. Fig. S2 Induction of pERF109::GFP nuclear fluorescence by Heterodera schachtii host invasion is disrupted in the coi1‐2 Arabidopsis mutant. Fig. S3 Root architecture of uninfected coi1‐2 and erf109 Arabidopsis plants differs from wild‐type Col‐0 plants. Fig. S4 DR5::GUS expression at the root tip does not differ between infected wild‐type Col‐0 and erf109 seedlings when auxin biosynthesis is inhibited only in the shoot. Fig. S5 Number of lateral roots in noninfected wild‐type Col‐0 and erf109 mutant seedlings is affected by l‐kyn treatment. Fig. S6 COI1‐mediated secondary root formation allows for maintenance of total root length despite primary root growth inhibition by Heterodera schachtii. Please note: Wiley is not responsible for the content or functionality of any Supporting Information supplied by the authors. Any queries (other than missing material) should be directed to the New Phytologist Central Office. [file NPH-237-807-s001.pdf]

New Phytologist

***New Phytologist* Supporting Information**

Article title: Root architecture plasticity in response to endoparasitic cyst nematodes is mediated by damage signaling

Authors: Nina Guarneri, Jaap-Jan Willig, Mark G. Sterken, Wenkun Zhou, M. Shamim Hasan, Letia Sharon, Florian M. W. Grundler, Viola Willemsen, Aska Goverse, Geert Smant, and Jose L. Lozano-Torres

Article acceptance date: 08 October 2022

The following Supporting Information is available for this article:

Fig. S1 Yuc split plate assay showing that ERF109 regulates local auxin biosynthesis at the nematode infection site.

Fig. S2 Induction of *pERF109::GFP* nuclear fluorescence by *Heterodera schachtii* host invasion is disrupted in the *coi1-2* Arabidopsis mutant.

Fig. S3 The root architecture of uninfected *coi1-2* and *erf109* Arabidopsis plants differs from wildtype Col-0 plants.

Fig. S4 *DR5::GUS* expression at the root tip does not differ between infected wildtype Col-0 and *erf109* seedlings when auxin biosynthesis is inhibited only in the shoot.

Fig. S5 The number of lateral roots in non-infected wildtype Col-0 and *erf109* mutant seedlings is affected by L-kyn treatment.

Fig. S6 COI1-mediated secondary root formation allows for maintenance of total root length despite primary root growth inhibition by *Heterodera schachtii*.

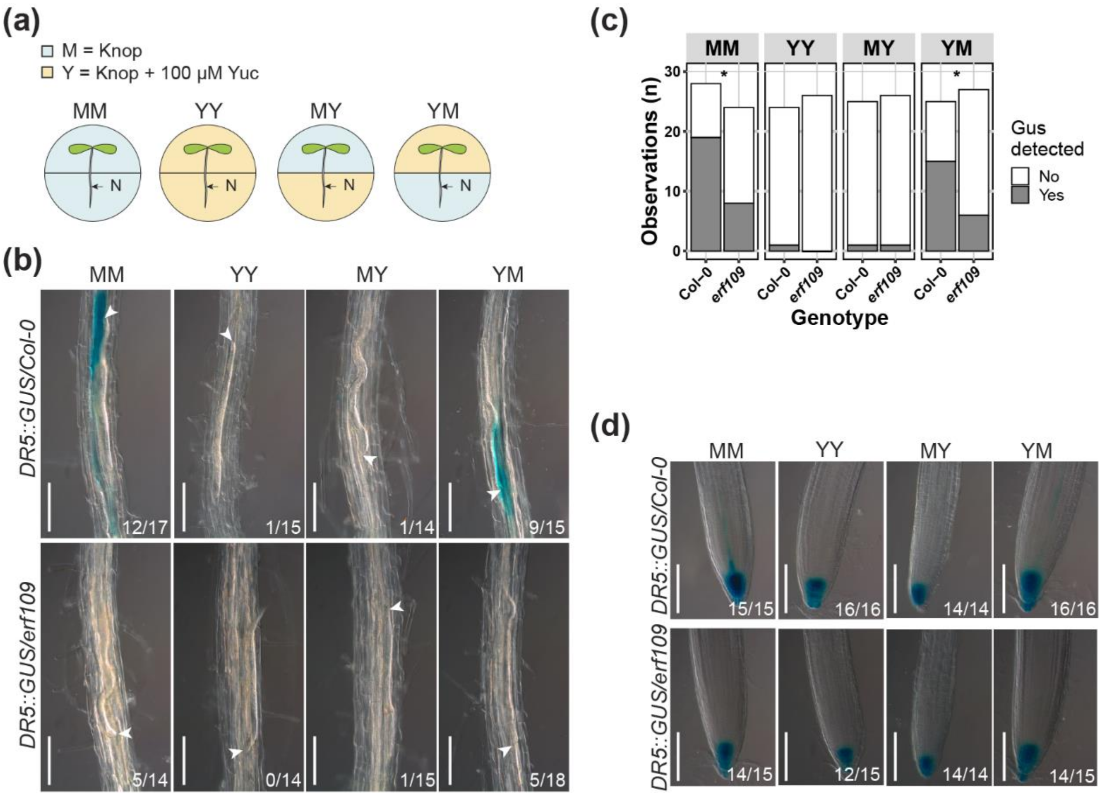

Fig. S1 Yuc split plate assay showing that ERF109 regulates local auxin biosynthesis at the nematode infection site. Four-day-old Arabidopsis Col-0 and *erf109* seedlings expressing the auxin *DR5::GUS* reporter were infected with 15 *Heterodera schachtii* second-stage juveniles (J2s). At 16 hours post inoculation, seedlings were transferred to treatment plates. Four treatment combinations were prepared: MM (modified Knop medium and 0.2% DMSO), YY (modified Knop medium, 100µM Yuc and 0.2% DMSO), MK (Yuc only in the root), YM (Yuc only in the shoot). At 3 days post inoculation GUS staining assay was performed for 4 hours and seedlings were imaged. Single-nematode infection sites were selected for observation. (a) Experimental design with Arabidopsis seedlings transferred to split plates with modified Knop medium either with or without Yuc. N = nematode. (b) *DR5::GUS* expression at nematode infection sites in wildtype Col-0 and *erf109* roots in the four different treatment combinations with or without Yuc applied to shoots and/or roots. (c) Number of observations with (Yes) or without (No) GUS staining at the nematode infection sites in roots of wildtype Col-0 and *erf109* plants. Statistical significance was calculated by a Pairwise Z-test (n=33, *, P<0.05). (d) *DR5::GUS* expression in the root tips of Col-0 and *erf109* roots. White arrowheads indicate the nematode

head. Frequencies at the bottom right corner indicate how many times GUS staining was observed in one of the three independent biological repeats of the experiment. Scale bar is 200 µm.

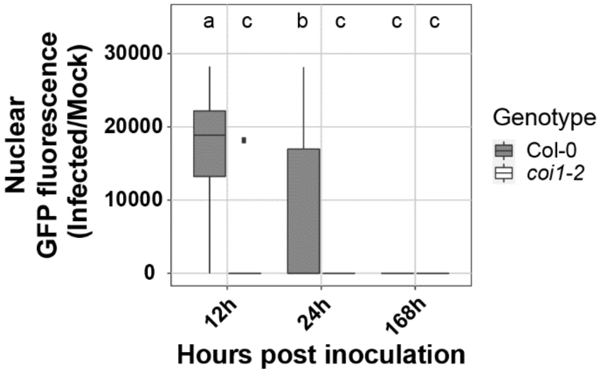

Fig. S2 Induction of *pERF109::GFP* nuclear fluorescence by *Heterodera schachtii* host invasion is disrupted in the *coi1-2* Arabidopsis mutant. Four-day-old Arabidopsis seedlings were either inoculated with 15 *H. schachtii* second-stage juveniles (J2s) or mock inoculated. At 12, 24, and 168 hpi seedlings were mounted in 10 µg ml^{-1} propidium iodide and then imaged. Nuclei were selected and the integrated density was measured using Fiji software. Ratio of the nuclear GFP fluorescence between infected and non-infected seedlings of wildtype Col-0 and *coi1-2* mutant. Significance of differences between fluorescent intensity in Col-0 and *coi1-2* roots over the different timepoints was calculated by Aligned Rank Transform for non-parametric factorial ANOVA followed by Tukey's HSD test for multiple comparisons (n=30, P<0.001). For boxplots, the horizontal line represents the median, the whiskers indicate the maximum/minimum range and the black dots represent the outliers. Difference in letters indicates statistically different groups.

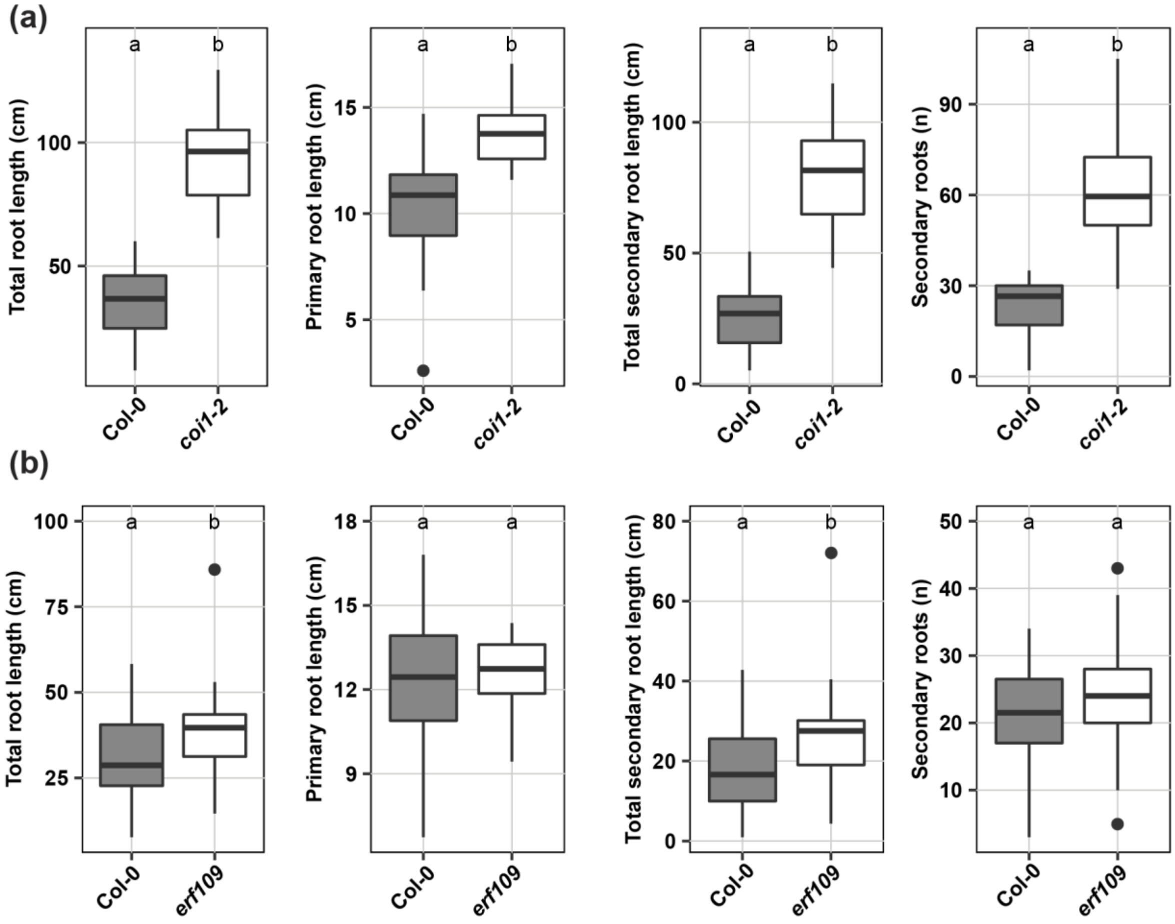

Fig. S3 The root architecture of uninfected *coi1-2* and *erf109* Arabidopsis plants differs from wildtype Col-0 plants. Scans of the roots of 16-day-old plants were made and the total root length was measured using WinRHIZO. For the experiment including *coi1-2* (a) the primary root was measured manually using ImageJ because of the complex root system of the mutant. For the experiment including *erf109* (b) the primary root length was automatically measured by WinRHIZO. Total secondary root length was calculated by subtracting the primary root length from the total root length. Data from three independent biological repeats of the experiment was combined. Significance of differences between genotypes was calculated by Student's T test (n=30, $P<0.05$). For boxplots, the horizontal line represents the median, the whiskers indicate the maximum/minimum range and the black dots represent the outliers. Different letters indicate statistically different groups.

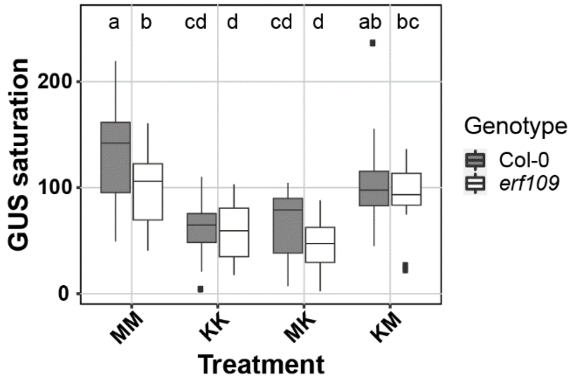

Fig. S4 *DR5::GUS* expression at the root tip does not differ between infected wildtype Col-0 and *erf109* seedlings when auxin biosynthesis is inhibited only in the shoot. Four-day-old wildtype Arabidopsis Col-0 and *erf109* mutant seedlings expressing the auxin *DR5::GUS* reporter were infected with 15 *Heterodera schachtii* second-stage juveniles (J2s). At 16 hours post inoculation, seedlings were transferred to treatment plates in a split-plate design. Four treatment combinations were prepared in split-plate assay: MM (modified Knop medium and 0.02%DMSO), KK (modified Knop medium, 10µM L-kyn and 0.02% DMSO), MK (L-kyn only in the root), KM (L-kyn only in the shoot). At 3 days post inoculation GUS staining was performed for 4 hours and seedlings were imaged. GUS saturation was measured as mean grey value using Fiji software. Data of two independent biological replicates was combined. Significance was calculated by ANOVA followed by Tukey's HSD test (n=20, $P<0.05$). For boxplots, the horizontal line represents the median, the whiskers indicate the maximum/minimum range and the black dots represent the outliers. Difference in letters indicates statistically different groups.

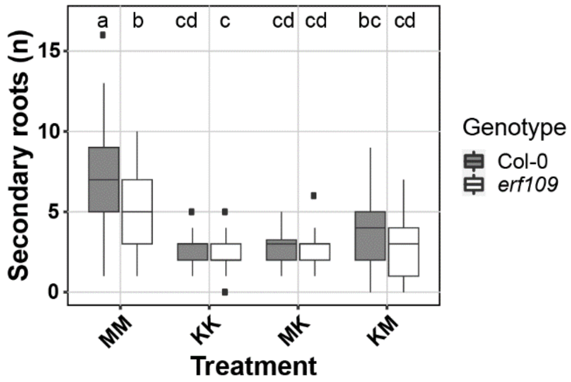

Fig. S5 The number of lateral roots in non-infected wildtype Col-0 and *erf109* mutant seedlings is affected by L-kyn treatment. Four-day-old Arabidopsis wildtype Col-0 and *erf109* seedlings were either infected with 15 *Heterodera schachtii* second-stage juveniles (J2s) or mock inoculated. At 16 hours post inoculation, seedlings were transferred to treatment plates in a split-plate design. Four treatment combinations were prepared: MM (modified Knop medium and 0.02% DMSO), KK (modified Knop medium, 10μM L-kyn and 0.02% DMSO), MK (L-kyn only in the root), KM (L-kyn only in the shoot). At 7 days post inoculation, scans were made of the root systems and the total number of secondary roots per plant was counted. Data of two independent biological repeats of the experiment was combined. Significance of differences in secondary roots between the different treatment combinations was calculated by ANOVA followed by Tukey's HSD test for multiple comparisons (n=43-45, $P<0.001$). For boxplots, the horizontal line represents the median, the whiskers indicate the maximum/minimum range and the black dots represent the outliers. Difference in letters indicates statistically different groups.

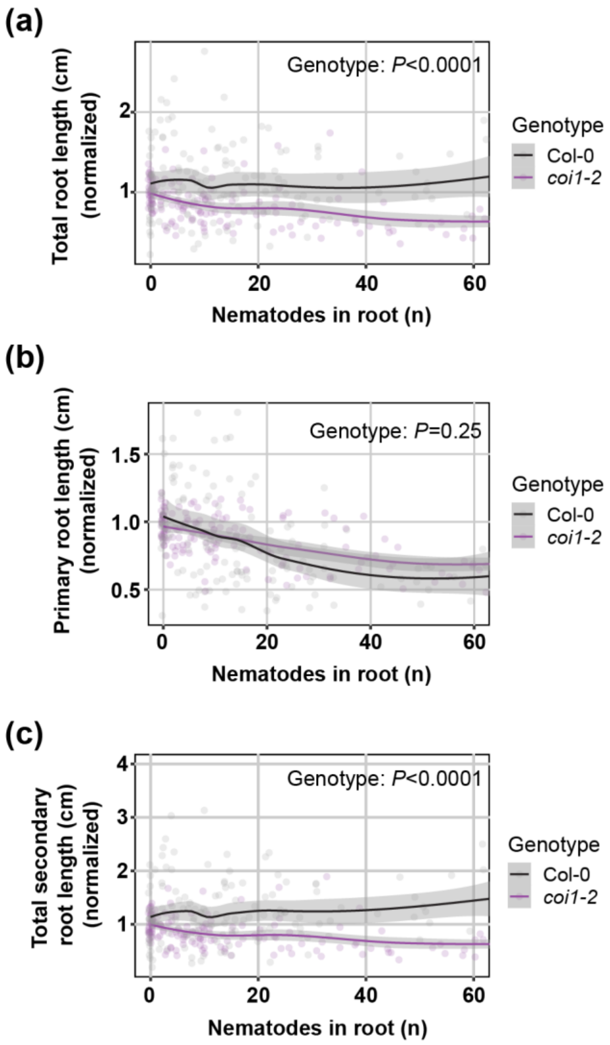

Fig. S6 COI1-mediated secondary root formation allows for maintenance of total root length despite primary root growth inhibition by *Heterodera schachtii*. Nine-day-old Col-0 and *coi1-2* Arabidopsis seedlings were inoculated with increasing *H. schachtii* densities ranging from 0 (mock) to 500 second-stage juveniles (J2s) per seedling. At 7 days post inoculation, scans were made of the root systems and the root length was measured using WinRHIZO. Total, primary, and secondary root length was normalized to the average respective component in mock-treated roots. Fuchsin staining was performed for counting the number of J2s that penetrated the roots. (a) Total root length per number of nematodes in the roots. (b) Primary root length per number of nematodes in the roots. (c) Total secondary root length per number of nematodes in the roots. Data from three independent biological repeats of the experiment was

combined. Significance of differences between genotypes was calculated by ANOVA (n=30). Grey area indicates the 95% confidence interval.
